# Sodium Selenite Improves Folliculogenesis in Radiation-Induced Ovarian Failure: A Mechanistic Approach

**DOI:** 10.1371/journal.pone.0050928

**Published:** 2012-12-06

**Authors:** Riham S. Said, Ahmed S. Nada, Ebtehal El-Demerdash

**Affiliations:** 1 National Center for Radiation Research and Technology, Atomic Energy Authority, Cairo, Egypt; 2 Department of Pharmacology and Toxicology, Faculty of Pharmacy, Ain Shams University, Cairo, Egypt; University of Medicine and Dentistry of New Jersey, United States of America

## Abstract

Radiotherapy is a major factor contributing to female infertility by inducing premature ovarian failure (POF). Therefore, the need for an effective radioprotective agent is evident. The present study investigated the mechanism of potential radioprotective effect of sodium selenite on radiation-induced ovarian failure and whether sodium selenite can stimulate *in-vivo* follicular development in experimental rats. Immature female Sprague-Dawely rats were either exposed to gamma-radiation (3.2 Gy, LD_20_), once and/or treated with sodium selenite (0.5 mg/kg), once daily for one week before irradiation. Follicular and oocyte development, apoptotic markers, proliferation marker as well as oxidative stress markers were assessed 24-h after irradiation. In addition, fertility assessment was performed after female rats became completely mature at two months of age. Sodium selenite significantly enhanced follicular development as compared to the irradiated group. Sodium selenite significantly reversed the oxidative stress effects of radiation that was evidenced by increasing in lipid peroxide level and decreasing in glutathione level, and glutathione peroxidase (GPx) activity. Assessment of apoptosis and cell proliferation markers revealed that caspase 3 and cytochrome c expressions markedly-increased, whereas, PCNA expression markedly-decreased in the irradiated group; in contrast, sodium selenite treatment prevented these alterations. Histopathological examination further confirmed the radioprotective efficacy of sodium selenite and its *in-vivo* effect on ovarian follicles’ maturation. In conclusion, sodium selenite showed a radioprotective effect and improved folliculogenesis through increasing ovarian granulosa cells proliferation, estradiol and FSH secretion, and GPx activity, whilst decreasing lipid peroxidation and oxidative stress, leading to inhibition of the apoptosis pathway through decreasing the expressions of caspase 3 and cytochrome c.

## Introduction

Premature ovarian failure (POF) is a heterogeneous disorder defined as cessation of ovarian function with elevated gonadotropins and low estrogen levels before or at the age of 40 [1]. It affects approximately one in 10,000 women by age of 20; one in 1,000 women by age of 30; one in 100 women by age of 40 [2]. Unexplained POF occurs in up to 1% of the world’s female population [3]; it is associated with loss of fertility, which in most cases is due to the absence of follicles, and in other cases, to the inability of remaining follicles to respond to stimulation [4]. It is well known that the ovaries contain a limited number of follicles; this number is about 200,000 at puberty and the progressive decrease with aging leads to only 400 follicles at the time of menopause [5]. Nevertheless, the currently used cancer therapies are often detrimental to fertility [6].

Radiotherapy, one of the most important cancer treatment modalities, relies on the generation and use of reactive oxygen species (ROS) to eradicate tumors [7], and in the process, non-target tissues are also damaged. The increase in ROS production in granulosa cells seems to have a deleterious effect on oocyte fertilization, embryo quality and implantation rate. Moreover, it seems that germ cells, in comparison with somatic cells, are more susceptible to oxidative stress [8], [9]. Additionally; radiotherapy is known to result in oocyte loss [10] and ovarian atrophy, combined with reduce follicle stores, leading subsequently to menstrual irregularities, ovarian failure and associated infertility.

Currently, the search for more effective radioprotectors has been intensified due to increased use of ionizing radiation in radiotherapy for the treatment of malignant tumors. Selenium (Se); an essential trace element; It is among the most well-known radioprotectors, and is necessary for the maintenance of various physiological processes [11]. Se is incorporated into the catalytic site of antioxidant enzymes, such as glutathione peroxidase (GPx), and is involved in cell growth and development by protecting cells against the toxic and damaging effects of ROS [12]. It is assumed that radiotherapy induces Se deficiency which possibly enhances radiation side-effects [13], [14]. As well, Paszkowski *et al*. [15] found a significant depletion of Se in the follicular fluid of women with unexplained infertility. In addition, Barrington *et al*. [16] demonstrated that idiopathic miscarriage is associated with Se deficiency. However, the biochemical mechanism through which Se prevents female reproductive disorders is not clear. One of the possibilities that needed further studying is modulation of oxidative stress and enhancement of the antioxidant system in the body. It was found that the synthesis of glutathione; an intracellular antioxidant, is a critical part of oocyte cytoplasmic maturation [16]. Indeed, the evidence from *in-vitro* studies indicated that Se is capable of improving the *in vitro* growth and maturation of the mouse preantral follicles [12]. Additionally, sodium selenite improved the *in-vitro* follicular development of immature mouse ovaries by reducing the ROS level and increasing the total antioxidant capacity and GPx activity [17]. Further, *in-vitro* studies using bovine granulosa cells demonstrated that Se significantly stimulated the proliferation of cells and enhanced estradiol production [18]. At the same time, it was registered that selenite had a radiosensitizing effect and increased the therapeutic index of radiation therapy for cancer cell lines [19], [20].

From the above, the effectiveness of Se in enhancing folliculogenesis *in-vivo* and the exact cellular mechanisms are not defined. Therefore, the present study was designed to examine whether Se has any significant role on radiation-induced ovarian-uterine dysfunction *in vivo* by studying its effects on different markers of oxidative stress, apoptosis, proliferation and folliculogenesis.

## Materials and Methods

### Chemicals

Sodium selenite, reduced glutathione (GSH), Ellman’s reagent [5,5-dithio-bis (2-nitrobenzoic acid); DTNB], and thiobarbituric acid (TBA) were purchased from (Sigma Chemical Co., St Louis, MO, USA). N-butanol, dipotassium hydrogen phosphate (K_2_HPO_4_), potassium di-hydrogen phosphate (KH_2_PO_4_), and trichloroacetic acid (TCA) were purchased from El-Nasr Chemical Co. (Egypt). Glutathione peroxidase kit was purchased from (Randox Laboratories, UK). All other chemicals and solvents were of the highest grade commercially available.

### Animals

The study was conducted according to the ethical guidelines (Ain Shams University, Egypt). Immature female Sprague-Dawely rats (23 days of age) were obtained from Nile Co. for Pharmaceutical and Chemical industries, Egypt. Rats were housed in an air-conditioned atmosphere, at a temperature of 25°C with alternatively 12-h light and dark cycles. Animals were acclimated for two weeks before experimentation. They were kept on a standard diet and water *ad libitum*. Standard diet pellets (El-Nasr, Egypt) contained not less than 20% protein, 5% fiber, 3.5% fat, 6.5% ash and a vitamin mixture.

### Gamma-radiation

Whole body gamma-irradiation was carried out using a Cesium (^137^CS) source, Gamma Cell-40 biological irradiator, at the National Centre for Radiation Research and Technology (NCRRT), Cairo, Egypt. The animals were exposed to a single dose of (3.2 Gy) gamma ray with a dose rate of 0.48 Gy/min. This dose represents the LD_20_ according to the study of Lee *et al*. [21]. The plastic boxes containing rats were positioned in a chamber fixed to the irradiation equipment.

### Experimental Design

Animals were divided into four groups (twelve animals per group) and treated for one week as follows; the first group acting as a control received saline (0.5 ml/100 g B.W., i.p.) once daily. The second irradiated group received saline (0.5 ml/100 g B.W., i.p.) once daily then exposed to a single dose of (3.2 Gy) whole-body irradiation with gamma ray. The third group was given sodium selenite (0.5 mg/kg, i.p.) once daily. The dose was chosen according to the study of Pontual *et al*. [22] and Cekan *et al*. [23]. The fourth group was administered sodium selenite (0.5 mg/kg, i.p.) once daily, and then exposed to a single dose whole-body irradiation. The second and the fourth groups were subjected to irradiation after 24-h of the last saline or sodium selenite injection. The control, irradiated, sodium selenite, and treated animals were tested daily for estrus cycles throughout the experiment using vaginal lavage techniques. Briefly, vaginal lavage was performed in the morning by flushing the vagina with 10 µl of distilled water and subsequently aspirated, then smeared onto a glass slide. The flushed vaginal fluid was fixed with 70% ethanol and examined microscopically using methylene blue stain. Twenty-four h after irradiation, blood samples were collected from the retro-orbital plexus and allowed to clot. Afterwards, rats were sacrificed; ovarian and uterine tissues were dissected, washed with ice-cold saline, and then, weighed.

### Tissue Collection and Processing

Serum was separated by centrifugation at 3000 g for 15 minutes and kept frozen at −80°C until assessment of 17 β-estradiol (E2) and follicle-stimulating hormone (FSH). Samples of ovarian and uterine tissues were homogenized at 1∶10 (w:v) in saline (PH 7.4) with an Ultra Turrax homogenizer after that the supernatant was obtained by centrifugation at 10,000 *g* for 15 minutes then, stored at –80°C until analysis of oxidative stress markers, including reduced glutathione (GSH), GPx activity and lipid peroxidation. In addition, further ovarian and uterine tissues were fixed in an appropriate buffer for light microscopical examination as well as immunohistochemical detection of proliferation marker, proliferating cell nuclear antigen (PCNA), and apoptotic markers, caspase 3 and cytochrome c.

### Circulating Levels of Serum E2 and FSH

To determine whether the fall in the number of growing follicles altered the functional maturation of the ovary, serum E2 and FSH were measured during the prepubertal period. An ELISA kit (DRG International, Inc., USA) was used for estimating the circulating levels of serum E2. As well, serum FSH measurements were performed by commercially available radioimmunoassay kit (rat FSH IRMA C.T., IBL International GMBH, Germany). The intra- and inter-assay coefficients of variation were found to be less than 9% and 10%, respectively for E2, and less than 3% and 8%, respectively for FSH. The minimum detectable concentration for E2 and FSH was 3.6 pg/ml and 0.2 ng/ml, respectively.

**Table 1:** Effect of sodium selenite injection (SS, 0.5 mg/kg, i.p.; once daily for 1 week) and/or whole body-irradiation on ovarian and uterine weights.

	Ovarian weight		Uterine weight	
Groups	mg	mg/100 g body weight	mg	mg/100 g body weight
Control	96.83±11.96	157.97±26.48	191.8±13.08	301.4±39.5
IR	58.85±12.65^a^	104.21±27.619^a^	112.5±27.48^a^	219.0±39.4^a^
SS	100.87±14.03^b^	168.12±31.34^b^	163.2±28.65^b^	293.1±53.2^b^
SS/IR	85.00±8.524^b^	149.38±35.92^b^	160.0±25.49^b^	287.1±24.6^b^

Data expressed as Mean ± SD.

a or b: Significantly different from control or radiation group, respectively at P<0.05 using one-way ANOVA followed by Tukey–Kramer as a post-hoc test.

### Measurement of Oxidative Stress

To determine GSH, 0.5 ml homogenate was added to a centrifuge tube with 0.5 ml of 10% TCA. The tubes were shaken gently and intermittently for 15 min, followed by centrifugation at 3000 *g* for 10 min. Aliquots of the resulting supernatant were added to a tube containing phosphate buffer and Ellman’s reagent then the absorbance was read at 412 nm within five min, according to Ellman’s method [24]. The results were expressed as µmol of GSH/g wet tissue. Lipid peroxidation was determined by estimating the level of thiobarbituric acid reactive substances (TBARS) measured as malondialdehyde (MDA), according to the method of Mihara and Uchiyama [25]. Briefly, the reaction mixture (0.5 ml homogenate +2.5 ml 20% TCA +1.0 ml 0.6% TBA) was heated for 20 min in a boiling-water bath, followed by cooling and addition of n-butanol with vigorous shaking. Afterward, the alcohol layer was separated by centrifugation at 2000 *g* for 10 min and the absorbance was measured at 535 nm. The results were expressed as nmol of MDA/g wet tissue using 1,1,3,3- tetraethoxypropane as standard.

**Figure 1:**
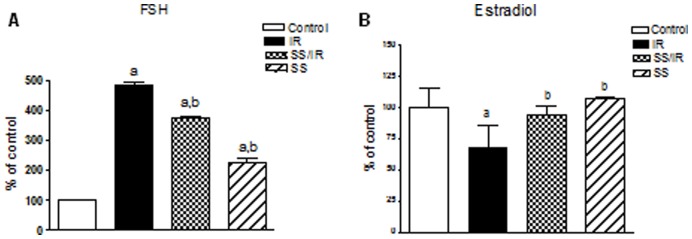
Circulating hormone levels. Changes in serum levels of FSH (A) and Estradiol (B), expressed as a percentage of control, after sodium selenite administration in *γ*-radiation subjected rats. Values are given as mean ± SD. a or b: Statistically significant from control or radiation group, respectively at P<0.05 using one-way ANOVA followed by Tukey–Kramer as a post-hoc test.

Furthermore, ovarian and uterine GPx activities were determined spectrophotometrically based on that of Paglia and Valentine [26]. GPx catalyses the oxidation of glutathione (GSH) by Cumene Hydroperoxide; in the presence of glutathione reductase and NADPH, the oxidized glutathione is immediately converted to the reduced form with a concomitant oxidation of NADPH to NADP^+^. The decrease in absorbance at 340 nm is measured. Specific activities were expressed as a unit/g wet tissue.

**Figure 2:**
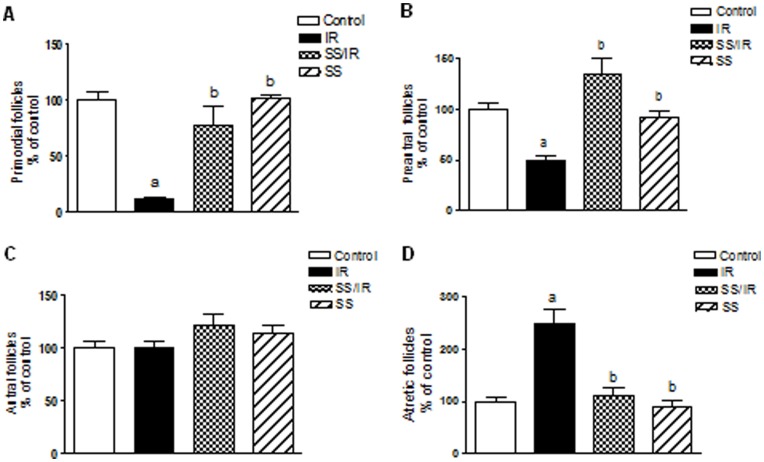
Morphometric analysis of ovarian follicle populations. Numbers of (A) primordial, (B) preantral, (C) antral and (D) atretic ovarian follicles was expressed as a percentage of control in each follicle type. Follicle counts were performed on histological sections as described in Materials and Methods. Bars represent the mean ± SD of at least three independent experiments. a or b: Statistically significant from control or irradiated group, respectively at P<0.05. Data were analyzed by one-way ANOVA followed by Tukey–Kramer as a post-hoc test.

### Histopathological Examination

The ovaries or uterus was fixed in 10% formalin overnight and embedded in paraffin. Serial sections of 4 µm thick were stained with hematoxylin and eosin for light microscopic histological examination. In all ovarian samples, the fifth cut was chosen to count the number of follicles and to evaluate follicular development using a digital video camera mounted on a light microscope (CX21, OLYMPUS, JAPAN). Follicles were classified as primordial if they contained an oocyte surrounded by flattened pregranulosa cells. Follicles were classified as pre-antral if they contained an oocyte with a visible nucleolus, more than one layer and less than five layers of granulosa cells and lacked an antral space. Follicles were classified as antral if they contained an oocyte with a visible nucleolus, more than five layers of granulosa cells and/or an antral space as described previously [27]. Atretic follicles were identified due to the presence of a degenerating oocyte or granulosa cells’ pyknosis [28].

**Figure 3:**
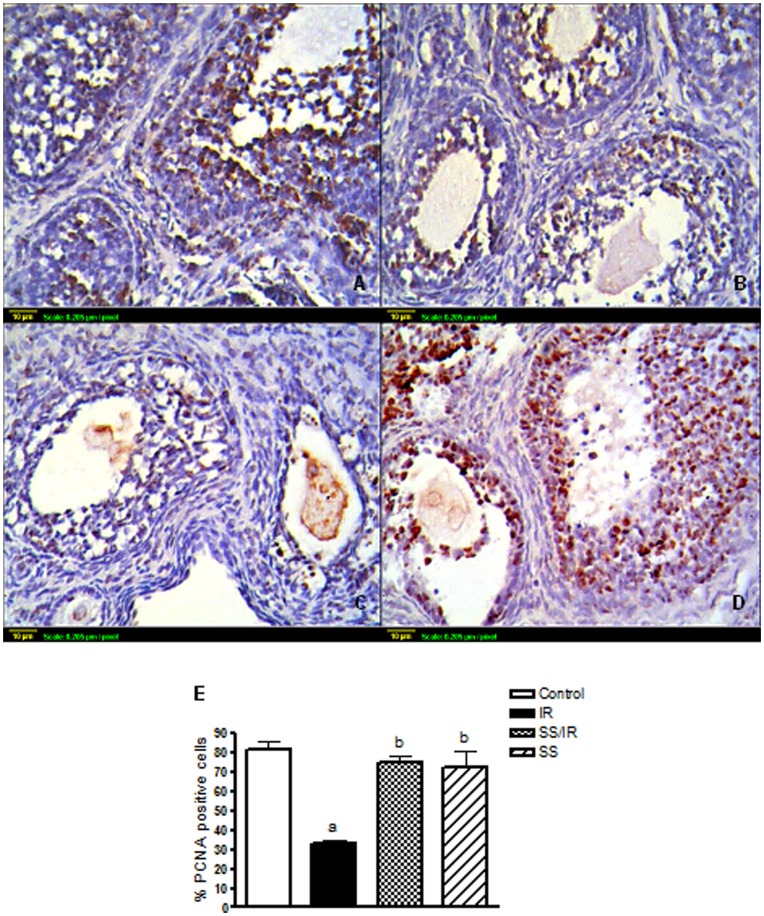
Follicular proliferation. Immunohistochemical localization of PCNA in ovarian follicles was studied 24-h after irradiation. (A) Expression of PCNA in ovaries of the control group. (B) Expression of PCNA in ovaries treated with sodium selenite (0.5 mg/kg) alone. (C) Expression of PCNA in ovaries exposed to *γ*-radiation (3.2 Gy). (D) Expression of PCNA in ovaries treated with sodium selenite (0.5 mg/kg) for one week before being exposed to *γ*-radiation (3.2 Gy). Scale bar, 10 *µ*m. (E) Quantitative image analysis for IHC staining expressed as a percentage of PCNA positive cells against the total number of granulosa cells across seven higher power fields (40×) for each rat section. Each column represents the mean ± SD of at least three independent experiments. a or b: Statistically significant from control or irradiated group, respectively at P<0.05 using one-way ANOVA followed by Tukey–Kramer as a post-hoc test.

### Immunohistochemistry

#### Proliferative marker

To determine whether the proliferative capacity of granulosa cells and thus the subsequent development of the growing follicles were modified in irradiated ovaries, immunohistochemical analysis of PCNA [29] was carried out. Paraffin embedded tissue sections of 3 µm thick were rehydrated first in xylene and next in graded ethanol solutions. The slides were then blocked with 5% bovine serum albumin in Tris buffered saline (TBS) for 2-h. Immunohistochemical analyses were performed by a standard streptavidin-biotin-peroxidase procedure. The sections were incubated with a mouse anti-PCNA monoclonal antibody (Thermo Fisher Scientific, Cat. No. MS-106-R7) overnight at 4°C. After rinsing thoroughly with TBS, the sections were incubated with a biotinylated goat anti-rabbit secondary antibody for 10–15 min, after that, the horseradish-peroxidase-conjugated streptavidin solution was added and incubated at room temperature for 10–15 min. Sections were then washed with TBS and incubated for 5–10 min in a solution of 0.02% diaminobenzidine (DAB) containing 0.01% H_2_O_2_. Counter staining was performed using hematoxylin, and the slides were visualized under a light microscope. The number of PCNA positively-stained cells over the total number of granulosa cells was counted in seven high-power fields (40×) using a digital video camera, then, the percentage of PCNA positive cells was calculated.

**Figure 4:**
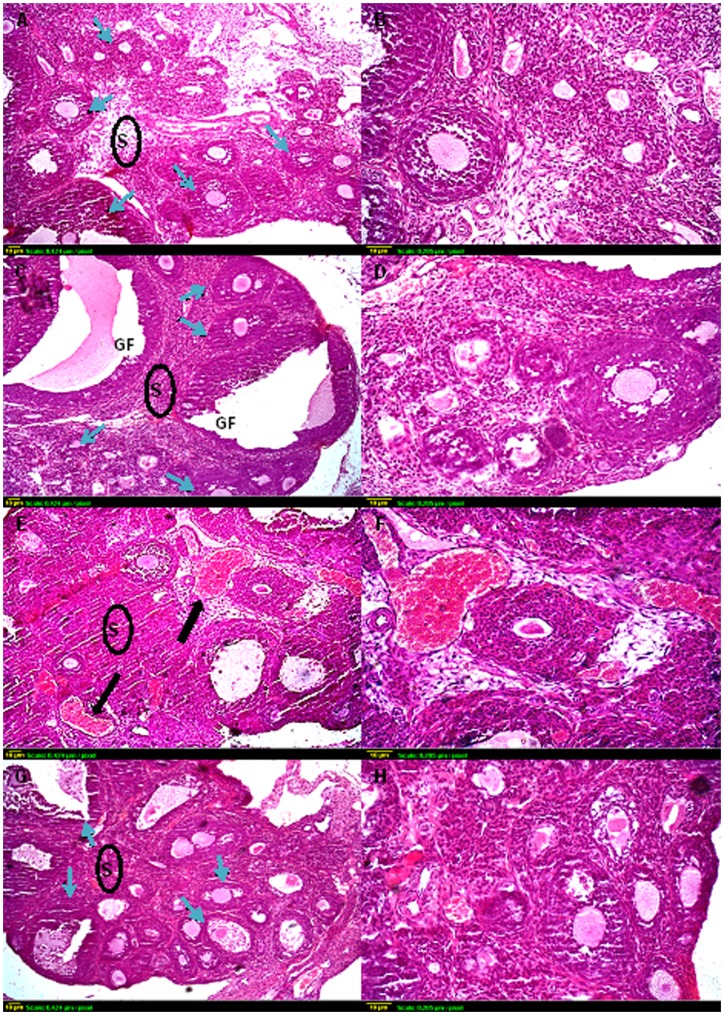
Representative photomicrographs of hematoxylin and eosin-stained ovarian tissue sections. Histological sections of control (A and B) and sodium selenite (C and D) treated ovaries exhibit similar organization, with different stages of growing follicles (blue arrows). Compared with controls, *γ*-irradiated ovaries (E and F) have few, if any, resting oocytes in the cortex with severe hemorrhage (black arrow). Many oocytes in small primary follicles are degenerating in irradiated ovaries. (G and H) Sections taken from ovaries of rats exposed to *γ*-irradiation and pre-treated with sodium selenite show the apparent normal structure of the ovary, with multiple types of ovarian follicles. Scale bar, 10 *µ*m. GF: Graffian follicle, S: Stroma.

#### Apoptotic markers

As previously-mentioned in PCNA assay, immunohistochemical analysis of apoptosis was carried out. Ovarian and uterine sections were incubated with primary antibody, which was a rabbit anti-active caspase 3 polyclonal antibody (abcam, Cat. NO. ab 2302) or a mouse anti-cytochrome c monoclonal antibody (Thermo Fisher Scientific, Cat. No. MS-1192-R7), using a biotinylated goat anti-rabbit, as secondary antibody. For negative controls, primary antibody was omitted.

**Figure 5:**
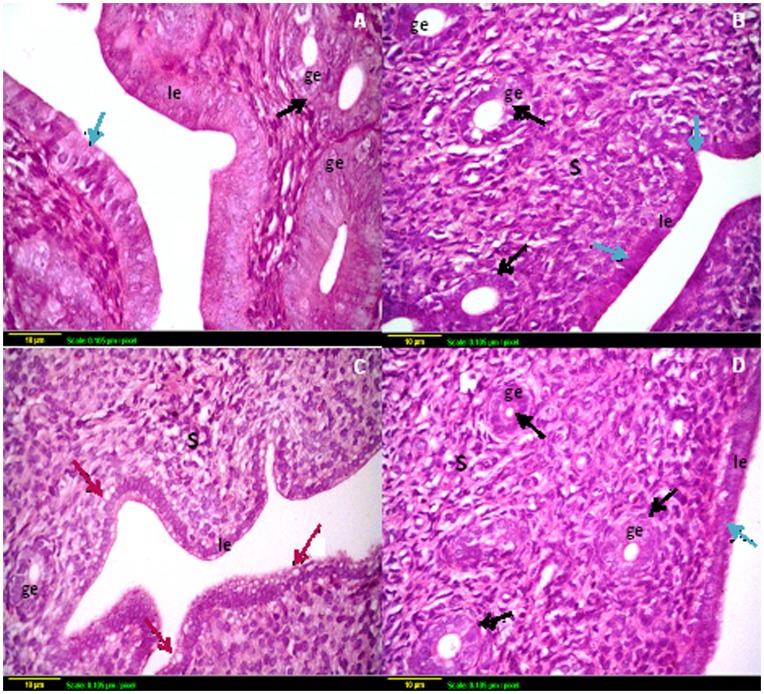
Representative photomicrographs of hematoxylin and eosin-stained uterine tissue sections. (A) Section taken from uterus of control rat and (B) Section taken from the uterus of sodium selenite treated rat show normal mucosal lining epithelium (blue arrow) with multiple glands (black arrow). (C) Section taken from the uterus of rats subjected to *γ*-radiation shows high degeneration of the mucosal epithelium with vacuole appearance (red arrow). (D) Section taken from the uterus of rats subjected to *γ*-radiation and pre-treated with sodium selenite shows regeneration of the glandular and luminal epithelium with intact structure. Scale bar, 10 *µ*m. le: luminal epithelium, ge: glandular epithelium, S: stroma.

Fractions of ovarian caspase 3 and cytochrome c DAB-positive immunoreactive areas were calculated automatically in seven high-power fields (20×), representing the percentage of immunopositive cells to the total area of the microscopic field using a digital video camera mounted on a light microscope (CX21, OLYMPUS, JAPAN). All steps for immunohistochemical evaluation were carried out using image analysis software (Image J, 1.46a, NIH, USA).

**Table 2:** Effect of sodium selenite (SS, 0.5 mg/kg, i.p.; once daily for 1 week) on oxidative stress markers in rats subjected to a single dose whole-body irradiation (3.2 Gy).

	GSH (µmol/g wet tissue)		GPx (U/g wet tissue)		MDA (nmol/g wet tissue)	
Groups	Ovary	Uterus	Ovary	Uterus	Ovary	Uterus
Control	4.04±0.48	5.01±0.43	5.36±1.46	2.01±0.23	84.8±22.1	96.2±10.11
IR	2.47±0.25^a^	4.40±0.09^a^	3.07±0.78^a^	1.24±0.43^a^	134.4±15.3^a^	275.0±64.55^a^
SS	3.39±0.17^b^	5.23±0.41^b^	5.89±0.56^b^	2.38±0.30^b^	82.7±14.3^b^	133.8±14.58^b^
SS/IR	3.93±0.64^b^	4.94±0.22^b^	4.87±0.48^b^	2.21±0.30^b^	86.4±8.9^b^	137.3±24.20^b^

Data expressed as Mean ± SD.

a or b: Significantly different from control or radiation group, respectively at P<0.05, using one-way ANOVA followed by Tukey–Kramer as a post-hoc test. Abbreviations: GSH, reduced glutathione; GPx, glutathione peroxidase; MDA, malondialdehyde.

### Fertility Assessment

Female rats from all groups (n = 5 in each group) were mated with age related, and sexually-experienced males at 60-day post natal (dpn) for up to 3 sexual cycles (3 estrus). One male was housed with a group of two or three females. Pregnant females at 14–15 d of gestation were isolated. Number of pregnant females and newborn pups were counted and kept with their mother until 2 dpn to check breastfeeding and eventual lethality.

### Statistical Analysis

Data are presented as the mean ± SD and were analyzed by one-way analysis of variance (ANOVA) followed by Tukey–Kramer as a post-hoc test. In addition, number of pups was compared using Kruskal-Wallis’s test followed by Dunn’s multiple comparisons as a post-hoc test. The 0.05 level of probability was used as the criterion for significance. All statistical analyses were performed using Instat version 3 software package. Graphs were sketched using GraphPad Prism (ISI® software, USA) version 5 software.

**Figure 6:**
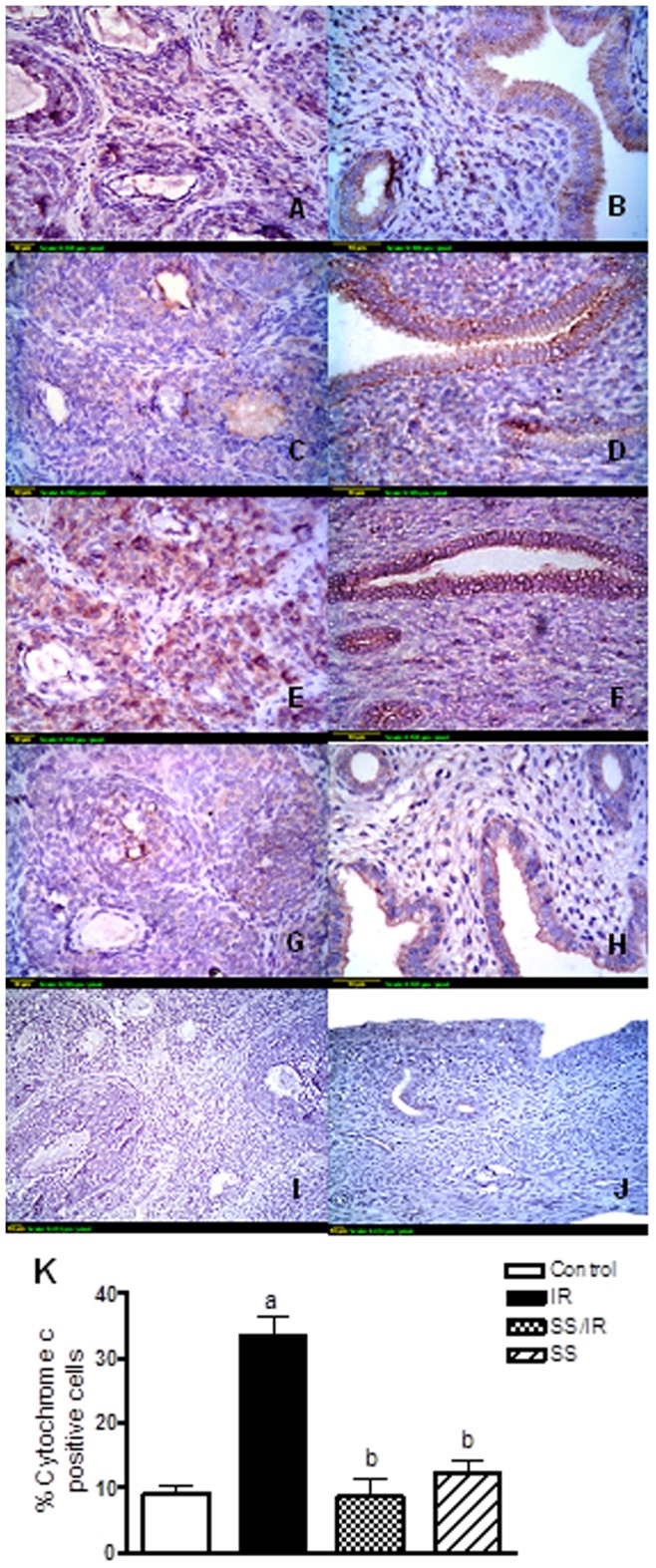
Immunohistochemical localization of cytochrome c. (A & B) Sections of ovaries and uterus of control rats show a minimal degree of cytochrome c expression. (C & D) Sections of ovaries and uterus treated with sodium selenite (0.5 mg/kg) alone show a minimal degree of cytochrome c expression. (E & F) Sections of ovaries and uterus exposed to *γ*-radiation (3.2 Gy) show extensive cytochrome c expression. (G & H) Sections of ovaries and uterus treated with sodium selenite (0.5 mg/kg) and exposed to *γ*-radiation (3.2 Gy) show limited cytochrome c expression. (I & J): Sections of ovarian and uterine negative controls omitting primary antibody. Scale bar, 10 *µ*m. (K): Quantification of ovarian cytochrome c staining represents the percent of immunopositive cells to the total area of the microscopic field (20×); averaged across 7 fields for each rat section. Each column represents the mean ± SD of at least three independent experiments. a or b: Statistically significant from control or irradiated group, respectively at P<0.05 using one-way ANOVA followed by Tukey–Kramer as a post-hoc test.

**Figure 7:**
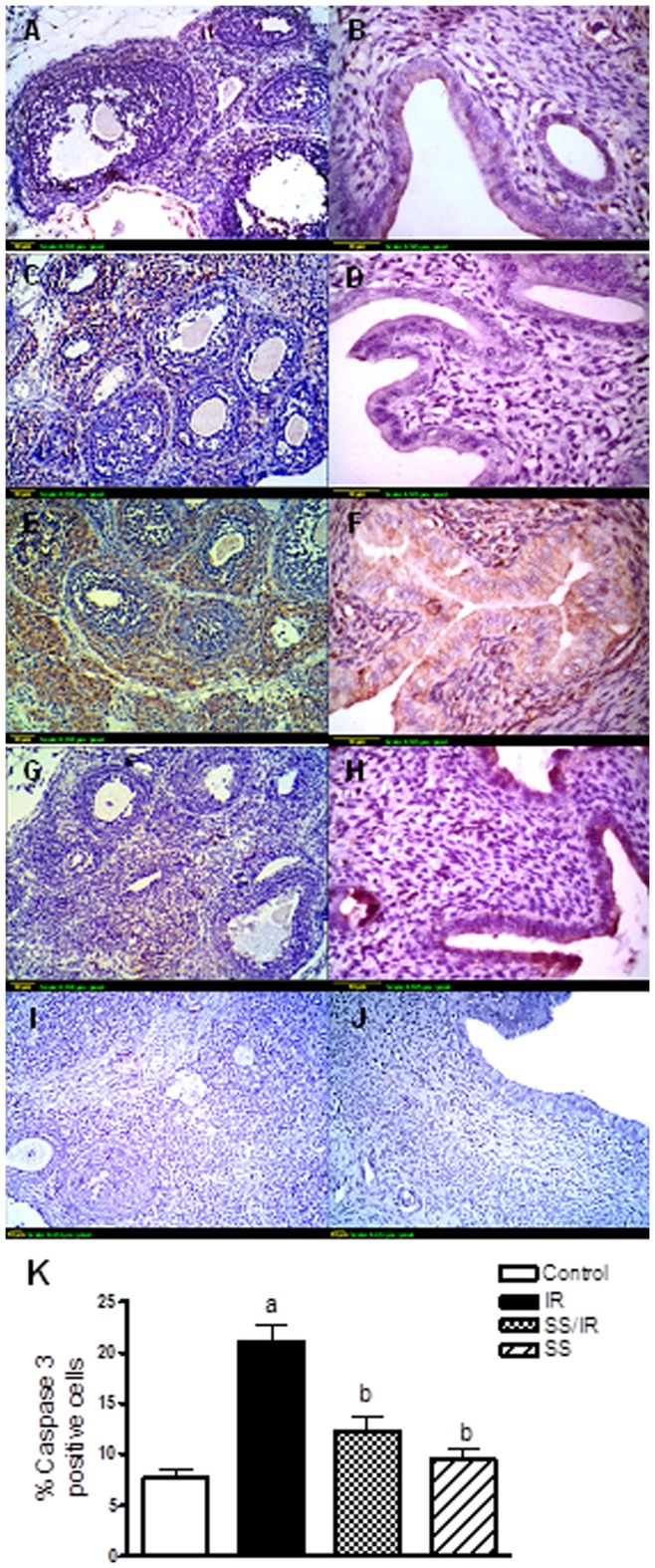
Immunohistochemical localization of caspase 3. (A & B) Expression of caspase 3 in ovaries and uterus of control groups shows a minimal degree. **(**C & D) Expression of caspase 3 in ovaries and uterus treated with sodium selenite (0.5 mg/kg) alone shows a minimal degree. (E & F) Expression of caspase 3 in ovaries and uterus exposed to *γ*-radiation (3.2 Gy) shows an extensive degree. (G & H) Expression of caspase 3 in ovaries and uterus treated with sodium selenite (0.5 mg/kg) for one week before being exposed to *γ*-radiation (3.2 Gy) was limited. (I & J): Ovarian and uterine negative controls omitting primary antibody. Scale bar, 10 *µ*m. (K): Quantification of ovarian caspase 3 staining represents the percent of immunopositive cells to the total area of the microscopic field (20×); averaged across 7 fields for each rat section. Each column represents the mean ± SD of at least three independent experiments. a or b: Statistically significant from control or irradiated group, respectively at P<0.05 using one-way ANOVA followed by Tukey–Kramer as a post-hoc test.

## Results

### Vaginal Smear and Organ Weight Changes

No differences in food consumption were seen between the groups of animals throughout the experimental schedule. Weights of ovarian and uterine tissues were compared after normalization to 100 g body weight. Animals exposed to radiation showed significant reduction in their ovarian and uterine weights, as compared to control group. Pre-treatment of animals with sodium selenite significantly counteracted effects of radiation and maintained sex organs’ weights comparable to that of the control group. Animals treated with sodium selenite alone did not show any significant difference from the control group (Table 1). Additionally, selenite-treated animals with or without *γ*-radiation exposures were undergoing estrus cycles, similar to controls; On the other hand, irradiated animals arrested at diestrus phase after 24-h of irradiation. However, after two months; before pregnancy; we observed that irradiation arrested the animals at estrus stage for a long time.

**Table 3:** Reproductive performance of control, irradiated and selenite treated females.

Groups	Fecundability	Fecundity
Control	100%	8.8
IR	20%	1.65^a^
SS	100%	8.75^b^
SS/IR	100%	8

Fecundability was expressed as a percentage of pregnant females among mated females. Fecundity was expressed as the number of pups per mated females. Fecundity values represent the median; they were compared by Kruskal-Wallis’s test followed by Dunn’s multiple comparisons as a post-hoc test, and differences were considered significant when P<0.05. a or b: Significantly different from control or radiation group, respectively at P<0.05.

### Circulating Hormone Levels

Serum FSH level of irradiated female rats showed a drastic increase of 4.84– folds (66±3.77 *vs.* 13.61±0.88 in controls). As shown in Fig. 1A, the serum FSH level was 22% lower in rats treated with sodium selenite before irradiation when compared with that of the irradiated group (51.26±1.38 *vs.* 66±3.77). Serum FSH level in the group of sodium selenite treatment alone was significantly higher by 127% (30.933±3.49 *vs.* 13.61±0.88 in control). In contrast, in irradiated females, serum E2 levels significantly decreased to 68% of the control values (21.1±5.26 *vs.* 30.88±4.944). Treatment of animals with sodium selenite before irradiation restored E2 level to near the basal levels as that of, the control group. Sodium selenite alone did not show any significant difference in serum E2 levels as compared to the control group (Fig. 1B).

### Folliculogenesis and Proliferation Markers

Morphometric analysis, during the immature period, showed that irradiated ovaries displayed a sequential reduction in the number of certain classes of follicles. The population of primordial follicles was reduced by 89% in irradiated ovaries (5±1 *vs*. 44±3.6 in controls), (Fig. 2 A). Additionally, radiation reduced the number of preantral follicles by 50% (8.66±1.52 *vs.* 17.33±2.08 in controls), (Fig. 2 B). Nevertheless, there was no significant difference in the population of healthy antral follicles between control and irradiated ovaries (Fig. 2 C). Conversely, the number of atretic follicles increased significantly in irradiated ovaries by 150% (15±2.64 *vs*. 5.99±1 in controls), (Fig. 2 D). Interestingly, treatment with sodium selenite before irradiation significantly increased the number of primordial and preantral follicles, and significantly decreased the number of atretic follicles as compared to the irradiated group. Treatment of animals with sodium selenite alone showed no significant effect on the follicles’ population as compared to the control.

Immunohistochemical detection of the proliferation marker, PCNA, demonstrated that follicles at the same stage of development were indeed at different states of maturation in control and irradiated ovaries. In control ovaries about half of the granulosa cells of growing follicles located in the center of the ovary were PCNA positive (Fig. 3 A). Treatment of rats with sodium selenite alone for one week showed PCNA expression levels similar to the control group (Fig. 3 B). In irradiated ovaries, the granulosa cells of all follicles; even the most developed, was mainly PCNA negative (Fig. 3 C). Irradiated ovaries pre-treated with sodium selenite, enhanced number of PCNA labeled granulosa cells of healthy pre-antral and antral follicles were positively-stained (Fig. 3 D). The immunohistochemical staining was quantified by counting the number of PCNA positive cells in seven high fields (40×), (Fig. 3 E).

### Ovarian and Uterine Histology

Ovarian sections from the control group stained with hematoxylin and eosin showed a normal ovarian structure which was characterized by different types of follicles, oocytes, and corpus luteum (Fig. 4 A & B). No abnormal histological alterations were observed in ovarian sections obtained from animals treated with sodium selenite alone as compared to the control group; however, these sections showed more follicular maturation (Fig. 4 C & D). In irradiated ovaries, the stromal tissue occupied most of the ovarian structure, which was characterized by severe hemorrhage and decreasing the number of growing follicles. In addition, very few primordial follicles were detected, and no primordial follicle stock was observed at the periphery of the irradiated ovary (Fig. 4 E & F). Administration of sodium selenite before irradiation preserved ovarian tissue from radiation-induced hemorrhage and maintained the follicular stock (Fig. 4 G & H).

Furthermore, uterine sections obtained from both control and sodium selenite treated groups showed a normal histological structure of mucosal lining epithelium and underlying lamina propria with glandular structure (Fig. 5 A & B). Animals exposed to radiation showed degeneration in the lining mucosal epithelial cells of the uterus which appeared as membrane blebbing; one of the features of apoptosis (Fig. 5 C). Interestingly, administration of sodium selenite before irradiation preserved the glandular and lining mucosal epithelium in the uterine structure (Fig. 5 D).

### Oxidative Stress Markers

Irradiation-induced oxidative stress in rat ovary and uterus were evaluated by assessing GSH, lipid peroxides levels as well as GPx activity. As shown in Table 2, radiation stimulated significant reduction of GSH level in ovarian and uterine tissues by 40% and 12%, respectively, as well as a significant increase in lipid peroxidation in ovarian and uterine tissues by 1.58 and 2.85 folds, respectively as compared to the control group. Treatment of animals with sodium selenite before exposure to irradiation afforded significant protection against radiation-induced oxidative stress and maintained levels of both GSH and lipid peroxides in ovarian and uterine tissues at near the control level. Furthermore, animals treated with sodium selenite alone did not show any significant alterations in GSH and lipid peroxide levels as compared to the control group (Table 2).

Further, GPx activities of ovarian and uterine tissues were significantly decreased after irradiation to 57.28% and 61.55%, respectively as compared to the control values. Neither ovarian nor uterine GPx activities were significantly different between control and sodium selenite alone treated animals. Pre-treatment of the irradiated group with sodium selenite significantly maintained GPx activities of both reproductive organs similar to that of the control group (Table 2).

### Apoptotic Markers

To determine whether radiation-induced apoptosis, immunolocalization of caspase 3 and cytochrome c were studied (Figure 6 & 7). It was found that ovarian granulosa and theca interstitial cells, and uterine epithelial cells in control rats showed minimal immunostaining for cytochrome c (Fig. 6 A & B) and caspase 3 (Fig. 7 A & B). Treatment of rats with sodium selenite alone showed cytochrome c (Fig. 6 C & D) and caspase 3 (Fig. 7 C & D) expression levels similar to the control group. Irradiation induced-marked increase in cytochrome c (Fig. 6 E & F) and caspase 3 (Fig. 7 E & F) expressions in ovarian granulosa and theca cells, and uterine epithelial cells with appearance of complete degeneration of the uterine mucosal lining epithelium associated with membrane blebbing, which was evident to the intense brown staining. However, pre-treatment of rats with sodium selenite significantly decreased the expressions of cytochrome c (Fig. 6 G & H) and caspase 3 (Fig. 7 G & H), when compared to the irradiated group. The immunohistochemical staining of cytochrome c and caspase 3 was quantified as a percentage of positive cells and the results are represented in (Fig. 6 & 7 K).

### Mating with Age Related Males

The reproductive capacity in terms of fecundability and fecundity was examined (Table 3). After female rats became completely adult, mating with age related normal males in the irradiated group resulted in significant decline in female fertility than control ones. Only one of 5 irradiated female animals was pregnant (20% vs. 100% in controls) and litter size was (1.62 vs. 8.8, pups for controls). In addition, irradiated females conceived pups with no morphological abnormality. While treatment of rats with sodium selenite one week before irradiation, preserved their fertility and increased the ability of females to become pregnant (100%) with litter size (8) pups/female. Female rats treated with sodium selenite only were led to (100%) pregnancy with litter size (8.75) pups/female, (Table 3).

## Discussion

Se radioprotective effects on several organs were previously demonstrated [30], [31]. However, its potential *in-vivo* radioprotective effect on the female reproductive organs is still uncertain. In the present study, the molecular mechanisms underlying the potential ovarian and uterine radioprotective effect of sodium selenite has been assessed by studying its effects on different markers of oxidative stress, apoptosis, proliferation and folliculogenesis in an experimental model of radiation-induced ovarian failure.

Ovarian failure manifests itself as hypergonadotropic hypogonadism resulting in amenorrhea and irreversible infertility [32]. In the present study, the irradiated females showed lower levels of serum E2 than those of the control group, whereas FSH concentration was extremely higher than control; these findings reflect the presence of typical ovarian failure. The increase in FSH level was attributed to abnormal steroid secretion from the ovaries. Pre-treatment of female rats with sodium selenite prevented the decrease in E2 level induced by irradiation. Our results are in accordance with an earlier study which showed that Se supplementation along with sodium arsenite treatment resulted in E2 level near the basal level, similar to the control group [33].

Morphometric analysis of the follicle population revealed that irradiation induced almost complete deletion of the primordial follicle pool within a time period of 24-h. In contrast, the other growing antral follicles remained unaffected. These findings, are in agreement with previous one [34], which indicated that oocytes contained in primordial follicles are particularly sensitive to ionizing radiation, whereas growing oocytes are relatively radio-resistant. Interestingly, in the current study, sodium selenite preserves primordial follicles’ stock, stimulates follicular maturation and minimizes the follicle depletion induced by radiation, suggesting the ovarian radioprotective effect of Se. Furthermore, injection of sodium selenite alone stimulated maturation of preantral follicles to more antral follicles.

Besides the morphometric analysis, the proliferation marker; PCNA was assessed. It was reported that in rat ovaries, the expression of PCNA was not detected in granulosa cells or oocytes in primordial follicles, but increased with the initiation of follicle growth [29]. In agreement with this study, as shown by PCNA staining, numerous granulosa cells of control ovaries in primary and preantral follicles were proliferating. In contrast, the proliferation of granulosa cells was markedly reduced in the ovarian follicles of the irradiated rats. It was reported that irradiation causes an elevation of p53 levels, mainly through a post-translation mechanism [35] and p53 regulates the expression of a number of downstream effector genes such as p21 [36]. The protein p21; a member of the family of cyclin-dependent kinase inhibitors, is a dual inhibitor of cyclin-dependent kinases (cdk) [37] and PCNA [38], [39], both of which are required for passage through the cell cycle. In the present study, pre-treatment of irradiated animals with sodium selenite markedly increased PCNA expression. Our results are in agreement with an earlier study which showed that Se at low concentrations may increase cell proliferation in HL-60 and U937 cells [40]. The pathway by which sodium selenite may stimulate cell proliferation is unclear, until now, but, according to the study of Zeng [41], cells supplemented with Se showed up-regulation of PCNA, cdk1, cdk2, cdk4, cyclin B and cyclin D2 mRNA levels. This finding suggests that Se directly or indirectly affects the function of basic transcriptional machinery in the cell. Furthermore, the present study demonstrated that treatment of animals with sodium selenite alone increased level of FSH significantly than control, which may be due to the stimulatory effect of Se on the dopamine level in the brain [42], [43], as dopamine has a stimulatory effect on gonadotrophin synthesis and secretion [44], [45]. So, it may be a possible mechanism by which sodium selenite stimulates folliculogenesis through increasing FSH concentration as the growing and mature follicles mark the onset of follicular maturation under the stimulus of FSH and LH [46]. The present study is the first one that confirmed the *in-vivo* follicular development effect of Se via promoting granulosa cells’ proliferation.

Besides the ovarian damage induced by irradiation, it was reported that uterine function may be impaired after radiation doses of 14 to 30 Gy, as a consequence of disruption of the uterine vasculature and musculature elasticity [47]. In the current study, uterine lining mucosal epithelium degeneration was observed after irradiation exposure, which may result from decreased ovarian E2 secretion induced by irradiation, as uterine growth depends on the ovarian E2 secretion [48]. Sodium selenite treatment prevented uterine degeneration which, implies that sodium selenite effectively attenuated the deterioration of radiation-induced uterine damage.

The deleterious effects of ionizing radiation in biological systems are mainly mediated through the generation of ROS in cells as a result of water radiolysis [49]. These ROS can induce oxidative damage to vital cellular molecules and structures, including DNA, lipids, proteins, and membranes [50], [51]. In this regard, the uterine endometrium degeneration which is associated with increased ROS production [52], as well as the decreased GPx activity in the follicular fluid, may be one of the major factors that is responsible for follicular regression [15], [53]. In the present study, it was found that acute irradiation exposure resulted in a significant increase in ovarian and uterine lipid peroxides accompanied with significant depletion of GSH level and decrease of the antioxidant enzyme activity of GPx as compared to the control group. The depletion in GSH contents after exposure to *γ*-radiation may be due to the reaction of GSH with free radicals resulting in the formation of thiol radicals that associated to produce oxidized glutathione [54]. Pre-treatment of rats with sodium selenite significantly counteracted the oxidative stress effect induced by radiation. These results support the important antioxidant role of Se in preventing lipid peroxidation and in protection of integrity and functioning of tissues and cells [55], [56]. Furthermore, these results confirmed the previous *in-vitro* study which hypothesized that sodium selenite improved follicular development by reducing ROS level and increasing GPx activity [17]. So, one of the possible mechanisms by which Se stimulates *in vivo* granulosa cell proliferation and improves follicular development is through increasing GPx activity, and decreasing oxidative stress and lipid peroxidation.

Besides the oxidative stress effect of radiation, apoptosis can be induced by ROS formed from ionizing radiation [57], [58]. The ROS induced mitochondrial membrane damage leading to the release of cytochrome c from the mitochondria into the cytosol which activates caspases and triggers apoptosis [59]. Since caspase 3, existing as a proenzyme, can become activated during the cascade of events associated with apoptosis [60]; therefore, its level is a good indicator of apoptosis. Ovarian granulosa cells play a key role in regulating ovarian physiology, including ovulation and luteal regression, which is a key to the fertility and pregnancy [61]. In ovarian failure, the ovarian follicles did not respond to high level of FSH caused by irradiation and did not secrete E2. Subsequently, FSH in this case stimulates ovarian follicles to apoptosis [62]. In fact, follicular atresia is apparently initiated by apoptosis of the granulosa cells [63]. In the present study, the assessment of apoptotic markers revealed that radiation significantly elevated the expressions of caspase 3 and cytochrome c in ovarian granulosa and theca cells, and uterine tissues as compared to control. In addition, sodium selenite could suppress apoptosis caused by ionizing radiation by decreasing the expressions of caspase 3 and cytochrome c. In agreement with these results, it was stated that a physiological concentration of Se in HT1080 cells increased cell proliferation and survival, and blocked the apoptotic signal [64]. So, another mechanism by which sodium selenite enhances the *in-vivo* follicular development could be through decreasing the apoptotic events induced by oxidative stress.

### Conclusions

The present study demonstrates that sodium selenite improves *in vivo* folliculogenesis and provides evidence for ovarian and uterine radioprotection. The mechanisms underlying these promising effects could be through increasing granulosa cells proliferation, E2 and FSH secretion, and GPx activity, while decreasing lipid peroxidation and oxidative stress leading to inhibition of apoptosis through decreasing the expressions of caspase 3 and cytochrome c.
